# Electrical Characterization of 3D Au Microelectrodes for Use in Retinal Prostheses

**DOI:** 10.3390/s150614345

**Published:** 2015-06-17

**Authors:** Sangmin Lee, Jae Hyun Ahn, Jong-Mo Seo, Hum Chung, Dong-Il “Dan” Cho

**Affiliations:** 1 Department of Electrical and Computer Engineering, ISRC/ASRI, Seoul National University, Seoul 151-742, Korea; E-Mails: sangmlee@snu.ac.kr (S.L.); brise98@snu.ac.kr (J.H.A.); callme@snu.ac.kr (J.-M.S.); 2 Department of Ophthalmology, College of Medicine, Seoul National University, Seoul 110-744, Korea; E-Mail: chungh@snu.ac.kr

**Keywords:** retinal prosthesis, 3D microelectrode, current stimulation, interface impedance, electrical durability

## Abstract

In order to provide high-quality visual information to patients who have implanted retinal prosthetic devices, the number of microelectrodes should be large. As the number of microelectrodes is increased, the dimensions of each microelectrode must be decreased, which in turn results in an increased microelectrode interface impedance and decreased injection current dynamic range. In order to improve the trade-off envelope between the number of microelectrodes and the current injection characteristics, a 3D microelectrode structure can be used as an alternative. In this paper, the electrical characteristics of 2D and 3D Au microelectrodes were investigated. In order to examine the effects of the structural difference, 2D and 3D Au microelectrodes with different base areas but similar effective surface areas were fabricated and evaluated. Interface impedances were measured and similar dynamic ranges were obtained for both 2D and 3D Au microelectrodes. These results indicate that more electrodes can be implemented in the same area if 3D designs are used. Furthermore, the 3D Au microelectrodes showed substantially enhanced electrical durability characteristics against over-injected stimulation currents, withstanding electrical currents that are much larger than the limit measured for 2D microelectrodes of similar area. This enhanced electrical durability property of 3D Au microelectrodes is a new finding in microelectrode research, and makes 3D microelectrodes very desirable devices.

## 1. Introduction

Retinal degenerative diseases such as age-related macular degeneration (ARMD) and retinitis pigmentosa (RP) result in progressive degeneration of the photoreceptors in the retina and eventually lead to complete blindness [1]. A number of tissue engineering and pharmaceutical treatment results to date have only managed to slow down of the disease progress and no method to stop this progress has been found yet [2,3]. Despite a near-total loss of the macular photoreceptors in the final stages of RP or ARMD, there are reports that the inner nuclear and retinal ganglion layers are partially preserved [4]. These results also support the notion that the approach of restoring vision by electrical stimulation of the surviving neurons using neural prosthetic devices may be viable as a vision-recovery methodology in retinal degenerative diseases [5]. Conventional retinal prosthesis systems consist of several parts, such as an external camera to receive visual information, signal processing circuits, stimulus microelectrode arrays (MEAs), and interconnection wires.

In electrical stimulation methods, MEAs are used to replace the functions of the degenerated natural photoreceptors by delivering electrical signals to the surviving inner nuclear and ganglion cell layers [6]. It is well known that at least 1000 pixel resolution is required to achieve facial recognition and reading [7]. Since the sensitive area in the retina is restricted to approximately 5 × 5 mm^2^, 1000 microelectrodes would need to be placed in that area. However, increasing the number of microelectrodes leads to high electrode impedances due to the reduction of the effective surface area of each unit, which in turn, limits the injection current amplitude [8]. In addition, the electrical impedance of the microelectrodes determines the dynamic range of the injection current, which is related to the operation voltage of the current stimulator IC. Recently reported current stimulators generally require high output voltage compliance, because the stimulators inject the biphasic current pulse with a maximum amplitude of several hundred μA and several tens of kΩ impedance into the microelectrodes and tissues. Consequently, this can generate quite high electrode voltages that may harm the tissues or damage the electrodes. Previous research has focused on various materials to enhance the electrical characteristics of microelectrodes [9]. As a different approach, we have been developing 3D microelectrodes since 2006. We have presented results on the fabrication [10,11], long-term biostability [12], and enhanced electrical/mechanical characteristics [12,13] of such devices. Other researchers have also reported results on 3D electrode technology [14]. 

In this paper, an experimental comparison between 2D and 3D Au microelectrodes is presented to evaluate their feasibility as high-resolution retinal prostheses. The comparisons between 2D and 3D microelectrodes are performed in an identical environment. The 3D Au microelectrode shows enhanced electrical impedance characteristics, allowing more electrodes in a given chip size as expected. However, this paper discovered a new phenomenon in that the 3D microelectrode can handle currents that exceed the theoretical limit, whereas the 2D microelectrodes show both electrical and physical failures near the theoretical limit. We name this novel property of microelectrodes electrical durability. 

## 2. Methods 

### 2.1. Fabrication of Microelectrodes

**Figure 1:**
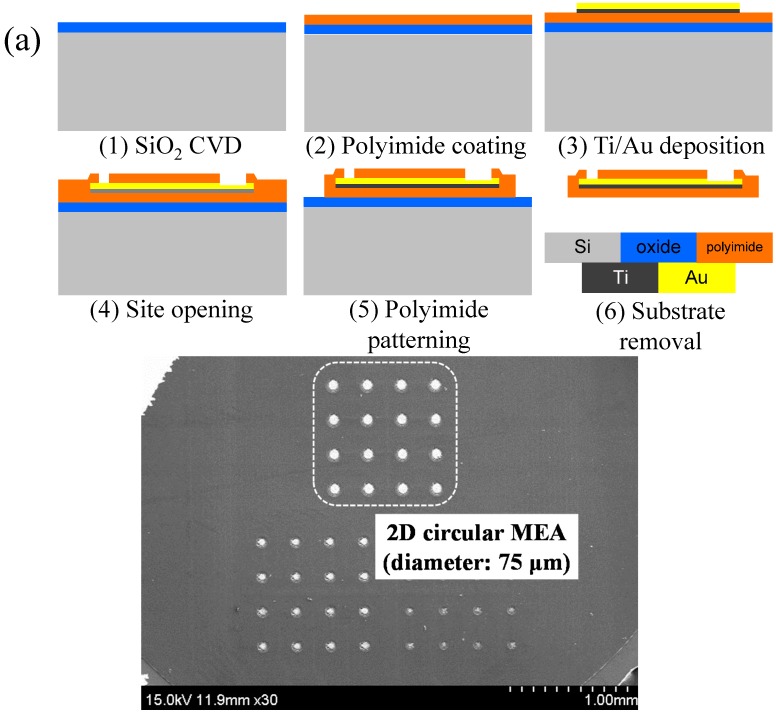

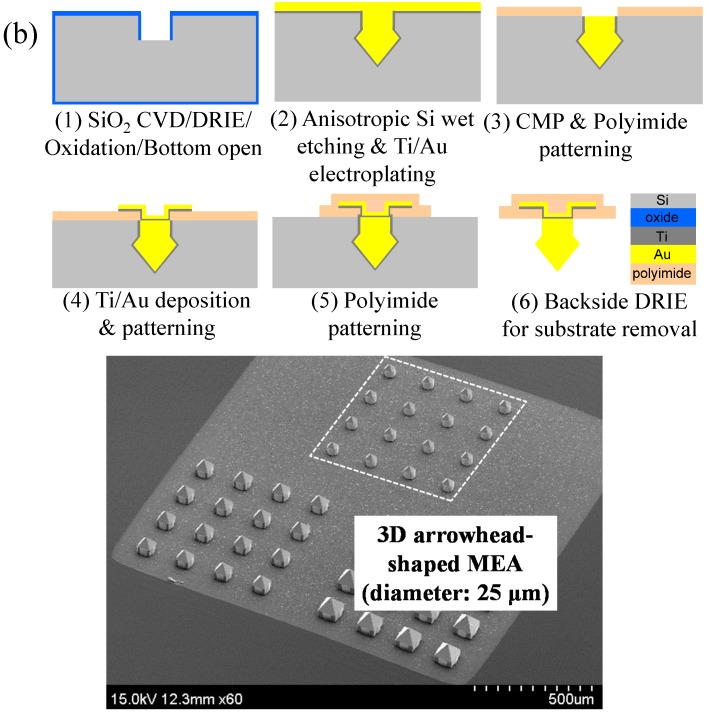
Fabrication processes and results. (**a**) 2D Au MEA; (**b**) 3D Au MEA.

Two types of MEAs were fabricated. The first type of MEA consists of 2D circular microelectrodes with an electrode diameter of 75 μm and an effective surface area of 4418 μm^2^. The second type of MEA consists of 3D arrowhead-shaped microelectrodes, which have a base electrode diameter of 25 μm and an effective surface area of 4802 μm^2^. The effective surface area of a 3D microelectrode can vary due to the electrode height of the cylindrical part. For the fabrication of 2D and 3D microelectrodes, we utilized the methods presented in [11,13,15], which use electroplating to fabricate the Au microelectrode structures. In this paper, the height of the cylindrical part is defined as 50 μm by the silicon deep-reactive-ion-etching (DRIE) process used. The fabrication processes and results of each MEAs are shown in Figure 1. The fabricated MEAs are wire-bonded to a printed-circuit board (PCB) to measure the electrical characteristics, such as the electrode-electrolyte interface impedance and the current injection limit.

### 2.2. Experimental Setup

**Figure 2:**
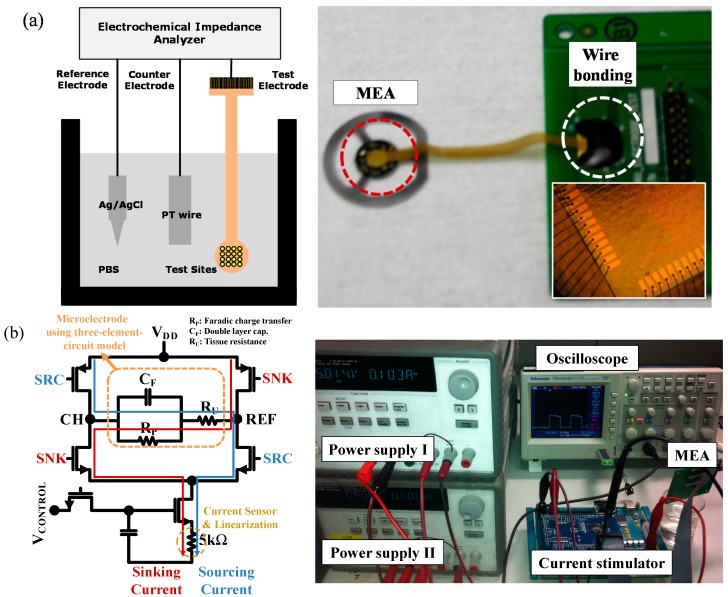
Experimental setup. (**a**) Interface impedance measurement (schematic of impedance measurement (left), fabricated MEA on PCB (right)); (**b**) Charge injection limit measurement (schematic of current stimulator (left), experimental setup (right)).

Figure 2 shows the experimental setups for measuring the electrode-electrolyte interface impedance and charge injection limit. It is necessary to measure the electrode-electrolyte interface impedance to determine the stimulation current level for the charge injection experiment. SI 1287 and SI 1260 impedance analyzers (Solatron, Schaumburg, IL, USA) were used to measure the impedance. Fabricated Au MEAs are dipped in a phosphate buffered saline (PBS) solution. Then, impedances are measured at 1 kHz, since the conventional current stimulation method is performed using biphasic currents of a 1 ms pulse duration. In order to measure the charge injection limit of each microelectrode, a current stimulator developed in our previous research [16] is used. The external current stimulator is a voltage controlled current source (VCCS), and it is a biphasic current stimulator with 5 V/12 V dual operation voltages. As shown in Figure 2a, when the SNK signal is “on”, the square-wave current waveform moves from REF to CH, and the current is measured as a voltage signal by using the built-in 5 kΩ load. After the intermediate duration of 1 ms, the SRC signal is turned “on” and the current waveform from CH to REF is measured and vice versa. From the built-in 5 kΩ load, the current passing through the microelectrode can be directly measured. 

## 3. Results

### 3.1. Interface Impedance

**Figure 3:**
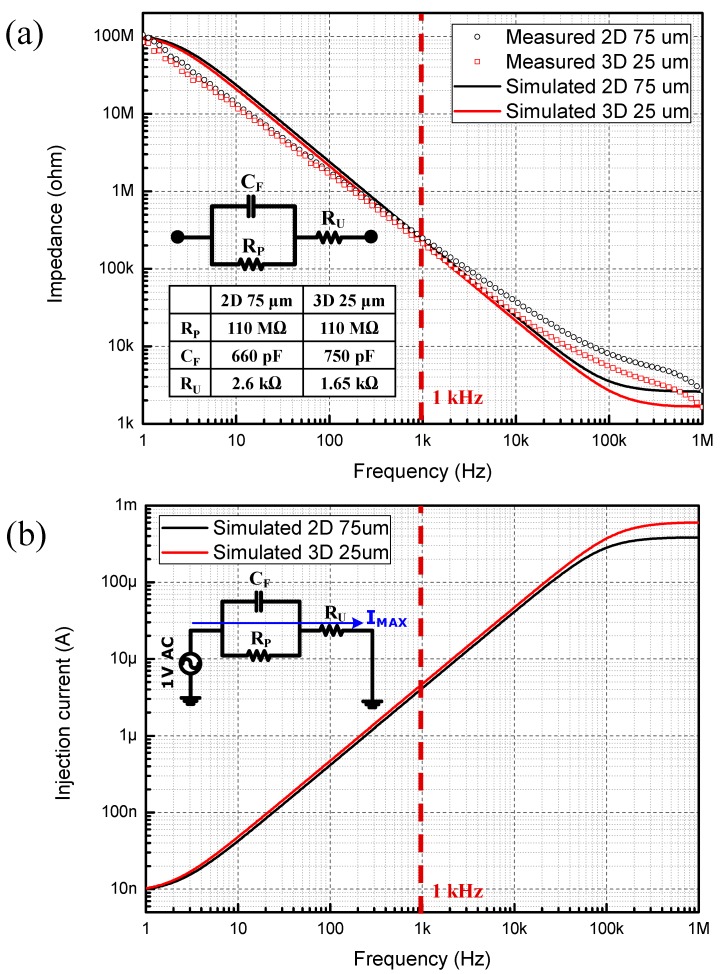
Electrical properties of 2D and 3D Au microelectrodes. (**a**) Measured interface impedance results (red and black circles) and parameterized interface impedance results using three-element-circuit model (red and black lines); (**b**) Theoretical current injection limit derived from three-element-circuit model using SPICE (at bias voltage 1 V).

In order to develop a high-resolution retinal stimulator device, it is necessary to examine the design consideration between MEAs and stimulator ICs. We have previously reported about the surface-area dependent electrical characteristics of 2D and 3D Au microelectrodes at a conference [13]. A theoretical amount of injection charge can be predicted by the value of the double-layer capacitance of each microelectrode using the three-element-circuit model [17]. The interface impedance measurement results are shown in Figure 3a. After the impedance measurement, each microelectrode is parameterized by the three-element-circuit model, and the maximum allowable current injection limit is simulated using the SPICE software [13]. Among the three-element-circuit model parameters, the most significant parameter that determines the injection current limit is the double-layer capacitance. The calculated double-layer capacitances of the 2D and 3D Au microelectrode in this paper are 660 pF and 750 pF, respectively. The results show that the 3D microelectrode which has a base diameter size of 25 μm has a similar interface impedance value as 2D microelectrode with a base diameter of 75 μm. The height of the cylindrical part of the 3D microelectrode is 50 μm. The effective surface areas of the 2D microelectrode with the diameter of 75 μm and 3D microelectrode with the diameter of 25 μm are 4418 μm^2^ and 4802 μm^2^, respectively. The estimated current injection limits of each microelectrode are also shown in Figure 3b. The measured impedance, the simulated impedance, and the simulated injection current for the 2D microelectrode are 248 kΩ, 241 kΩ, and 4.15 μA, respectively. The corresponding values for the 3D microelectrode are 212 kΩ, 211 kΩ, and 4.71 μA, respectively. The similar results are shown for 2D and 3D microelectrode, which are anticipated for similar surface areas.

### 3.2. Electrical Durability

As shown in Figure 3b, the estimated theoretical current injection limits of the 2D Au microelectrodes and 3D Au microelectrodes at a bias voltage of 1V are 4.15 μA and 4.71 μA, respectively. The external current stimulator used in this paper is a voltage controlled current source (VCCS) developed previously by us, which is implemented by a high-voltage (5 V/12 V dual operation voltage), 0.35 μm, Bipolar-CMOS-DMOS (HV-0.35-BCDMOS) process [16]. The operation voltage of the current stimulator used in the experiment is set at 12 V. The calculated maximum injection current limits through the 2D and 3D microelectrodes are about 49.8 μA (= 4.15 μA × 12) and 56.5 μA (= 4.71 μA × 12), respectively. 

**Figure 4:**
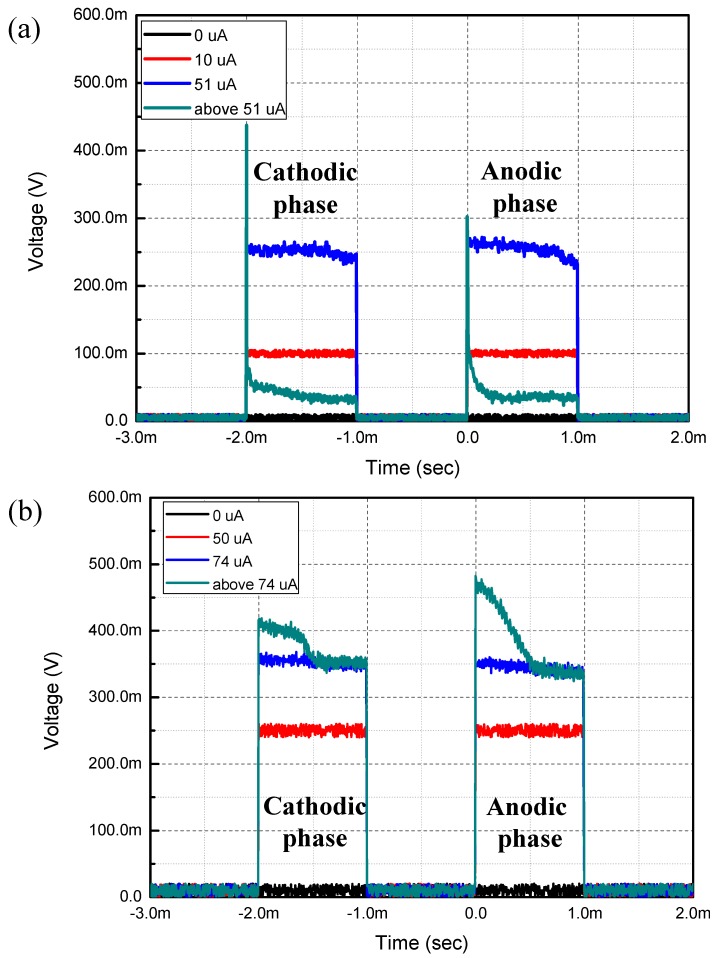
Time domain output of electrical durability evaluation. (**a**) 2D Au microelectrode; (**b**) 3D Au microelectrode.

**Figure 5:**
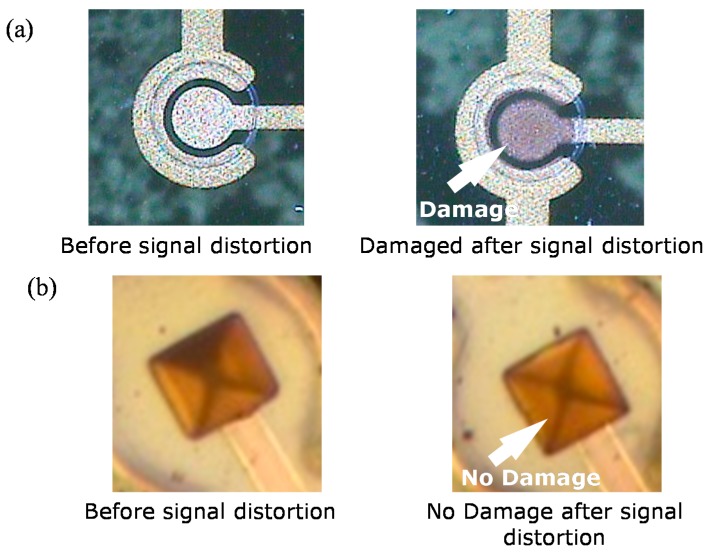
Microscopic image of microelectrode before and after signal distortion. (**a**) 2D Au microelectrode; (**b**) 3D Au microelectrode.

The current injection experiment results of both 2D and 3D Au microelectrodes are shown in Figure 4. The first part is the cathodic phase and the second part is the anodic phase. As the current flow passing through the microelectrode and the built-in 5 kΩ load increases, the measured voltage signal also increases. For the 2D Au microelectrodes, an injection current below a certain limit is delivered well (red line in Figure 4a). However, as the injection current is increased to approximately 51 μA (which is slightly over the calculated limit of 49.8 μA), the time domain output curve starts to distort (blue line in Figure 4a). As the injection current exceeds the limit, the time domain output curve becomes fully distorted (green line in Figure 4a), indicating a physical failure. In fact, the surface of the 2D Au microelectrode becomes damaged, as shown in Figure 5a. The same procedure is performed for the 3D Au microelectrode. For the 3D Au microelectrode, however, the time domain output curve is not distorted until the injection current is approximately 74 μA, which far exceeds the calculated theoretical limit of 56.5 μA. Even at a current level above 74 μA the output is not fully distorted. In fact, as shown in Figure 5b, the 3D Au microelectrode is not damaged even when it is exposed to a current level above the limit. Therefore, compared to the 2D microelectrodes, the 3D microelectrodes can handle approximately 30.9% more stimulating current without damaging the electrode surface.

**Figure 6:**
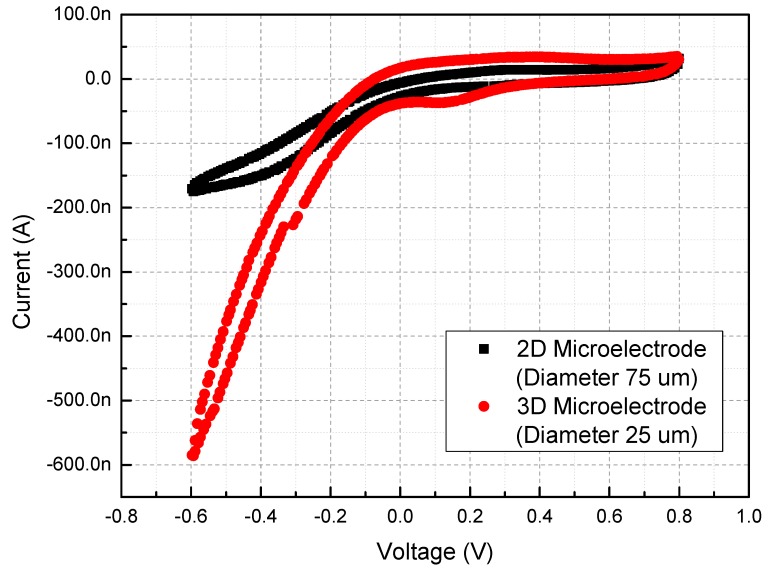
Cyclic voltammetry results of 2D and 3D Au microelectrode.

This is a new finding, which can be explained in part by the cyclic voltammetry results shown in Figure 6. The cyclic voltammetry measurements are performed with a voltage sweep from −0.6 V to 0.8 V and the slew rate of 50 mV/s. The charge storage capacity (CSC) is determined by integrating the area under either the cathodic or anodic sweep in the cyclic voltammetry plot within the water window. The obtained value indicates the maximum charge that can be injected by a reversible surface process by an electrode [18]. The CSCs of 2D and 3D Au microelectrode are calculated from the CV curve using the time integral of the current during the full potential cycle between the sweep ranges, and the measured results give CSCs of 2.67 μC/cm^2^ and 5.69 μC/cm^2^, respectively. Because of the slow slew rate compared to the actual current stimulation method, the cyclic voltammetry results should not be used to quantify the accurate charge injection limit, but rather to explain the new observed phenomenon. The electrical durability can be influenced by the volume density, inner-structural morphology, material porosity of the electrode material, or other factors. This is in contrast to the electrode impedance which seems to depend only on the total exposed surface area. We believe the result reported in this paper, that 3D Au microelectrodes have increased electrical durability compared to the 2D Au microelectrodes is very important for electrode-based stimulation research. Therefore, we are pursuing a more extensive study on this phenomenon to further quantify and generalize the electrical durability results, which will be reported in near future. *In vivo* experiments are also planned and will be reported at a later time.

## 4. Conclusions

The results in this paper show that 3D Au microelectrodes are more advantageous than 2D Au microelectrodes to fabricate high-resolution retinal prostheses, which require highly-densified microelectrodes and high electrical durability. Interface impedances were measured and similar stimulation current dynamic ranges were obtained for both 2D and 3D Au microelectrodes with similar effective surface areas. This implies that an MEA with 3D microelectrodes can achieve higher spatial resolution than an MEA with 2D microelectrodes. Furthermore, the three-element-circuit model is used to evaluate the circuit parameters, and to estimate the theoretical current-injection limit of the 2D and 3D microelectrodes. For the 2D Au microelectrodes, the dynamic range of the injection current between theoretical value and experimental value is matched within the error range of 2.4%. However, for that of the 3D Au microelectrodes, the measured value exceeds the theoretical value by 30.9%. This phenomenon is due to the enhanced electrical durability of the 3D Au microelectrodes, which indicates that the 3D Au microelectrodes can handle currents that exceed the simple area factor. The results of this research can be used to define requirements for high-resolution retinal prosthetic systems as well as other neural stimulation applications.
